# Prospective Bi-Centric Real-World Outcomes of Upadacitinib in Biologic-Experienced Patients with Crohn’s Disease

**DOI:** 10.3390/diseases14020054

**Published:** 2026-02-01

**Authors:** Janina Lüke, Clara Zippel, Phil-Robin Tepasse, Frank Lenze, Markus Strauss, Arne Bokemeyer, Joost Buskermolen, Tina Schomacher, Julia Fischer, Jonel Trebicka, Richard Vollenberg

**Affiliations:** 1 Department of Neurology, Herz-Jesu-Krankenhaus Münster-Hiltrup, 48165 Münster, Germany; 2 Department of Medicine B, Gastroenterology, Hepatology, Endocrinology and Clinical Infectious Diseases, University Hospital Muenster, 48149 Münster, Germany; 3 Department of Department of Internal Medicine II, Gastroenterology and Hepatology, St. Barbara-Klinik, Hamm-Heessen, 59073 Hamm, Germany; 4 Department of Cardiology I-Coronary and Peripheral Vascular Disease, Heart Failure Medicine, University Hospital Muenster, 48149 Münster, Germany; 5 Department of Cardiology, Faculty of Health, School of Medicine, University Witten/Herdecke, 58448 Witten, Germany; 6 Department of Gastroenterology, Diabetology and Palliative Medicine, Bonifatius Hospital Lingen, 49808 Lingen, Germany

**Keywords:** Crohn’s disease, Janus kinase (JAK) inhibitors, upadacitinib

## Abstract

Background: The efficacy of upadacitinib in patients with Crohn’s disease (CD) has been shown in pivotal randomized controlled trials. However, real-world data is needed to assess its effectiveness and safety in routine clinical care with biologic-experienced patients. This study aimed to evaluate the clinical and endoscopic efficacy, patient-reported outcomes (PROs), and safety of upadacitinib in biologic-experienced patients with CD in a real-world setting. Methods: This prospective bi-centric real-world study enrolled 28 anti-TNF-experienced patients with CD receiving upadacitinib 45 mg daily for 12 weeks (induction), followed by 30 mg daily maintenance through week 52. Primary endpoints included endoscopic response (≥50% SES-CD reduction or ≥2-point decrease from baseline for baseline SES-CD ≤ 4) and clinical remission (Harvey–Bradshaw Index [HBI] ≤ 4). Secondary endpoints included endoscopic remission, clinical response (HBI decrease ≥ 3 points), and quality of life (IBD-Disk). Statistical analysis used the Wilcoxon signed-rank test with 95% confidence intervals (CIs). Results: Median patient age was 37 years; 75% had ≥3 prior biologic failures. Clinical remission rates (HBI) were 59% (95% CI: 41–75%) at week 12, 44% (95% CI: 27–63%) at week 26, and 53% (95% CI: 29–76%) at week 52. Endoscopic response rates were 48% (95% CI: 44–52%) at week 26 and 46% (95% CI: 21–72%) at week 52. Endoscopic remission was achieved in 43% (95% CI: 40–48%) at week 26 and 27% (95% CI: 10–57%) at week 52. Clinical response (HBI) improved progressively from 65% at week 2 to 71% at week 52. Quality of life, as assessed by the IBD-Disk, showed significant improvement: Reduced Disease Burden (defined as a decrease of 70% or a CED-Disk Score of ≤15) was observed in 33% of patients at week 12 and 35% at week 52. Median SES-CD decreased from 9 points (IQR: 6–17) at baseline to 5 points (IQR: 1–12, *p* = 0.005) at week 52. Adverse events occurred in 11% of patients (4% lymphopenia, 7% skin disease), with no serious adverse events or deaths. Conclusions: Upadacitinib demonstrates significant clinical and endoscopic efficacy in biologic-experienced, anti-TNF-pretreated patients with CD, achieving remission rates comparable to or exceeding those of the pivotal trials despite a highly refractory population (75% with ≥3 prior biologic failures). The favorable safety profile supports upadacitinib as an important therapeutic option in sequential treatment of refractory CD.

## Simple Summary

Crohn’s disease is a chronic inflammatory bowel condition that often requires biologics to control inflammation. However, some patients do not respond adequately to these treatments. Upadacitinib is a newer oral medication that works through a different mechanism than traditional biologics. This study examined whether upadacitinib is effective and safe in patients with Crohn’s disease who had failed previous biologic treatments. We followed 28 patients receiving upadacitinib for one year and measured their clinical symptoms, intestinal healing, and quality of life. More than half of patients achieved clinical remission, and the medication improved intestinal inflammation in nearly half of the patients. Most importantly, upadacitinib was well-tolerated with minimal side effects. These findings suggest that upadacitinib represents a valuable treatment option for patients with difficult-to-treat Crohn’s disease and could help expand therapeutic options in clinical practice.

## 1. Introduction

Crohn’s disease (CD) belongs to the group of inflammatory bowel diseases (IBD) and is characterized by chronic intestinal inflammation, which can lead to complications such as fistulas, abscesses, and stenoses [1,2]. Therapeutic goals have evolved over recent years with the availability of modern treatment options, shifting away from clinical remission alone towards steroid-free remission accompanied by normalization of quality of life and reduction in complications [3]. According to the STRIDE II recommendations, mucosal healing is increasingly discussed as a therapeutic goal [3,4]. Novel mechanisms of action and new therapeutic agents are needed to achieve this objective.

While biologics have substantially advanced the therapeutic management of CD in recent years, the therapeutic options have recently been broadened by the introduction of small molecule therapy with upadacitinib. The JAK-mediated activation of signal transducers and activators of transcription (STATs) in T cells constitutes an important pathogenic factor in CD [5,6]. The efficacy and safety of upadacitinib has been demonstrated in pivotal randomized controlled trials (RCTs; U-EXCEL, U-EXCEED, U-ENDURE), leading to the approval of CD therapy in 2023. However, real-world data are necessary to evaluate its effectiveness and safety in routine clinical care, where patient populations and treatment settings are more heterogenous. Furthermore, the previously mentioned RCTs primarily included biologic-naïve patients, while the use of upadacitinib in patients with CD pretreated with multiple biologics was not among the primary study objectives [2,7,8].

Against this background, we conducted a bi-centric prospective real-world trial investigating the therapeutic efficacy of upadacitinib in biologic-experienced, anti-TNF-pretreated patients with CD during the induction and maintenance phases through week 52. Our study aimed to assess clinical and endoscopic treatment response as well as patient-reported outcomes.

## 2. Materials and Methods

### 2.1. Trial Design and Oversight

In this prospective bi-centric real-world phase study (data collection period 04/2023–10/2025), patients aged ≥ 18 years with a diagnosis of CD who were anti-TNF-experienced and had planned initiation of upadacitinib therapy were enrolled. The indication for therapy was not determined as part of the study but included prior primary non-response, secondary loss of response, or side effects from existing immunosuppressive therapy. Before therapeutic switching, a baseline ileocolonoscopy was performed and laboratory markers (fecal calprotectin, inflammatory markers) were determined. The Harvey–Bradshaw Index (HBI) and the Visual Analog Scale (IBD-Disk) were used to quantify clinical impairment due to CD.

**Figure 1:**
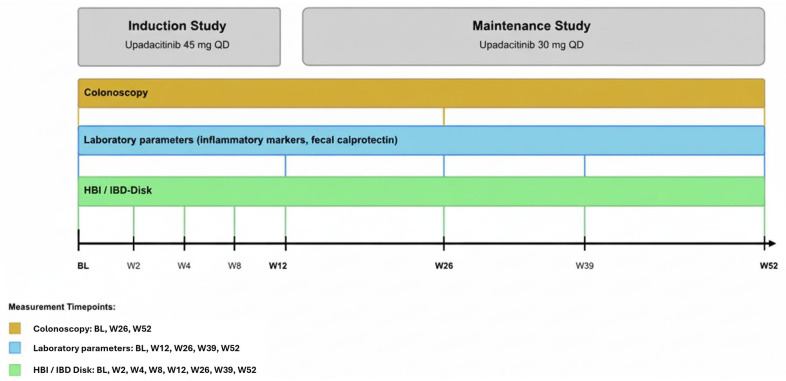
Study Flow chart. Depiction of assessment time points for clinical and endoscopic treatment response during the induction and maintenance phases. QD (quaque die), once daily; HBI, Harvey–Bradshaw Index; IBD-Disk, inflammatory bowel disease disk; BL, baseline; W, week.

Endoscopic assessment of intestinal inflammatory activity was performed at weeks 26 and 52 using the Simple Endoscopic Score for Crohn’s Disease (SES-CD). This scoring system evaluates four parameters (ulcer size, ulcerated and affected surfaces, and stenosis) across five bowel segments (ileum, right colon, transverse colon, left colon, and rectum), with each segment rated on a scale of 0 to 3. The total score ranges from 0 to 56, with higher values reflecting greater disease severity [9]. The SES-CD was obtained by the examining gastroenterologist during colonoscopy and verified by a central reader (R.V./P.-R.T.) part of the study based on photographic documentation. For clinical disease activity assessment, the HBI captures general well-being, abdominal pain, the number of liquid/soft stools, abdominal mass, and extraintestinal manifestations (arthralgia, uveitis, erythema nodosum, aphthous ulcers, pyoderma gangrenosum, anal fissures, new fistula, abscess) [10]. A higher score value is associated with clinical impairment. The Visual Analog Scale (IBD-Disk) contains ten component subscales: abdominal pain, bowel control, interpersonal interactions, education/work, sleep, energy, emotions, body image, sexuality, and joint pain. Total score ranges from 0 to 100, with higher scores indicating impaired health-related quality of life [11]. Clinical treatment response based on HBI and IBD-Disk was quantified at weeks 2, 4, 8, 12, 26, 39, and 52 after initiation of upadacitinib therapy. Laboratory parameters (inflammatory markers, fecal calprotectin) were determined at weeks 12, 26, 39, and 52 (Figure 1).

Failure of therapy was defined as inadequate response to or unacceptable side effects from therapy. Analysis was performed according to a per protocol approach. In this analysis, only patients who remained enrolled in the study were evaluated at each respective assessment timepoint. Patients who withdrew prematurely from the study were consequently not included in the analysis.

Apart from prednisolone, no other concomitant immunosuppressive therapies were used. In patients receiving concomitant prednisolone pulse therapy, prednisolone tapering was intended. Depending on the disease course, this had to be individually adapted.

### 2.2. Efficacy and Safety Evaluations

The primary study endpoints of endoscopic response were evaluated at weeks 26 and 52, and clinical remission (HBI) was assessed during induction (weeks 2, 4, 8, 12) and maintenance phase (weeks 26, 39, 52). Endoscopic response was defined as a reduction of more than 50% in the SES-CD score from baseline (or, for patients with a baseline SES-CD of ≤4, a decrease of ≥2 points), while clinical remission using the Harvey–Bradshaw Index (HBI) was defined as an HBI score of ≤ 4.

Secondary endpoints for induction and maintenance therapy included endoscopic remission (SES-CD of 4 or less, a decrease of at least 2 points from baseline) and clinical response in terms of HBI score (decrease in HBI score ≥ 3 points), as previously defined [12]. Additionally, patient-reported outcomes (PROs) were assessed using the IBD-Disk score; since validated thresholds have not yet been established for the IBD-Disk score, these were defined analogously to established criteria in IBD research. Improved Disease Burden (IDB) was defined as a reduction in the IBD-Disk score of ≥30% from baseline, and Reduced Disease Burden (RDB) was defined as a score of ≤15 points or a reduction of ≥70% from baseline. Additionally, patients with a high disease burden were classified based on IBD-Disk scores greater than 30, while patients with low impairment in daily life were classified based on IBD-Disk scores of 30 or less (Supplemental Table S1) [13].

#### Statistical Analysis

For pairwise analysis of changes between baseline and the respective time points, the Wilcoxon signed-rank test for paired samples was used for non-normally distributed samples. Median, interquartile range (IQR), and minimum–maximum values are presented. Patients with treatment response are reported as percentages with the corresponding confidence interval (Wilson CI). Safety data were summarized descriptively. Statistical significance was determined using two-tailed tests at a significance level of *p* < 0.05. Data analysis was conducted using SPSS version 26 (IBM, Armonk, NY, USA), GraphPad Prism software (version 9, GraphPad Software, La Jolla, CA, USA), the R package, and RStudio (RStudio Team (2015). RStudio: Integrated Development for R. RStudio, Inc., Boston, MA, USA, http://www.rstudio.com/ (accessed on 2 February 2024).

## 3. Results

### 3.1. Patient Characteristics

This bi-centric prospective real-world study enrolled 28 patients with CD (two centers: University Hospital Muenster, Germany and Barbara Hospital Hamm, Germany) who received induction therapy with upadacitinib 45 mg daily for 12 weeks according to the approved labeling. During the maintenance phase, all patients were reduced to a maintenance dose of 30 mg/day.

At treatment initiation, patients with CD had a median age of 37 (range 25–64) years. The median disease duration was 15 (range 9–18) years, and 13 (46%) participants were male. All enrolled CD patients had prior biological therapy with anti-TNF (100%; 28/28). Furthermore, 75% (21/28) had three or more prior biologics, 14% (4/28) had two prior biologics, and 11% (3/28) had one prior biological therapy, suggesting a cohort with extensive prior treatment exposure. Additionally, 54% (15/28) had prior therapy with azathioprine/6-mercaptopurine and 21% (6/28) had prior therapy with methotrexate (MTX) (Table 1).

### 3.2. Induction/Maintenance Phase After Initiation of Upadacitinib: Primary and Secondary Endpoints (Per Protocol Analysis)

As primary endpoints, patients with CD showed an endoscopic response in 48% (95% CI: 44–52%) of patients at week 26 after treatment initiation with upadacitinib at a maintenance dose of 30 mg/day, and in 46% (95% CI: 21–72%) of patients at week 52. Clinical remission (defined by HBI) was observed at the end of the induction phase at week 12 in 59% (95% CI: 41–75%) of patients, in the maintenance phase at week 26 in 44% (95% CI: 27–63%) of patients, and at week 52 in 53% (95% CI: 29–76%) of patients (Table 2).

As secondary endpoints, endoscopic remission was achieved in 43% (95% CI: 40–48%) of patients with CD at week 26 and in 27% (95% CI: 10–57%) at week 52.

With respect to further clinical assessment, clinical response (defined by HBI) during the induction phase was observed in 65% of patients (95% CI: 46–81%) at week 2, in 68% (95% CI: 48–83%) at week 4, in 72% (95% CI: 52–86%) at week 8, and in 78% (95% CI: 59–86%) at week 12. In the maintenance phase, clinical response was observed in 52% (95% CI: 33–70%) of patients with CD at week 26, in 65% (95% CI: 41–83%) at week 39, and in 71% (95% CI: 62–72%) at week 52 (Table 2).

According PROs using the IBD-Disk, 65% (95% CI: 60–67%) of patients demonstrated an IDB, and 38% (95% CI: 37–43%) showed RDB consistent with a patient-reported remission profile at week 2 during the induction phase. At week 12, the proportion of patients with CD with IDB was 70% (95% CI: 65–71%) and RDB 33% (95% CI: 32–39%); at week 26, 48% (95% CI: 45–52%) for IDB and 24% (95% CI: 24–31%) for RDB; and at week 52, 53% (95% CI: 47–58%) for IDB and 35% (95% CI: 33–43%) for RDB.

An IBD-Disk score of less than 30, indicating a low disease burden before initiation, was observed in 9 (12%) patients, in 15 (56%) patients at week 12, and in 13 (77%) patients at week 52 among those with IBD (Supplemental Table S1).

### 3.3. Efficacy Analyses, Quality of Life, and Safety Outcomes

Analyzing the efficacy of upadacitinib treatment in this real-world cohort of patients with CD, endoscopic assessment revealed significant improvement: the median SES-CD score was 9 points (IQR: 6–17) before treatment initiation and significantly dropped to 6 points (IQR: 2–12, *p* = 0.014) at week 26, and to 5 points (IQR: 1–12, *p* = 0.005) at week 52. Furthermore, patients showed a significant clinical response rate (defined by HBI) during the induction and maintenance phase: a significant clinical treatment response was already observed at week 2 after treatment initiation (HBI 9 (IQR: 5–14) versus 5 (IQR: 0–7), *p* < 0.001) and showed significant improvements at all further assessment points during the induction phase (weeks 2, 4, 8, 12) and in the maintenance phase (Table 3). Laboratory assessment showed that fecal calprotectin was at a median of 350 µg/g (IQR: 100–1280 µg/g) before upadacitinib initiation and dropped to 184 µg/g (IQR: 58–809, *p* = 0.157) at week 12 after initiation, 289 µg/g (IQR: 88–723 µg/g, *p* = 0.181) at week 26, 114 µg/g (IQR: 29–870 µg/g, *p* = 0.05) at week 39, and 214 µg/g (IQR: 17–449 µg/g, *p* = 0.875) at week 52 (Table 3).

Correspondingly, PROs were assessed using the IBD-Disk. Clinically significant improvements compared to baseline before treatment initiation were also observed at all time points examined. Already at week 2 of the induction phase, a significant improvement from 53 points (IQR: 23–73) to 23 points (IQR: 4–47, *p* < 0.001, Table 3) was observed. Before treatment initiation, the areas of education/work, sleep, energy, and arthralgias were particularly severely impaired by the underlying disease. After treatment initiation, significant improvements occurred in all subcategories as early as 2 weeks (Table 3).

Concomitant prednisolone therapy (≤20 mg prednisolone/day) was present at treatment initiation in 15 (54%) patients (Table 3), and at week 52, only 1 of 16 patients (6.25%) received steroids reflecting modern IBD management.

Evaluating the reason for discontinuation of upadacitinib during the study period, treatment was discontinued in three patients due to adverse events (one patient with lymphopenia, two patients with skin diseases, Table 4). No severe adverse events (SAEs) occurred, and there were no deaths. In seven patients (25%), therapy was discontinued by week 52 due to non-response (Table 5).

There was a presentation of the proportion of patients with a response, including the Wilson 95% confidence interval.

**Figure 2:**
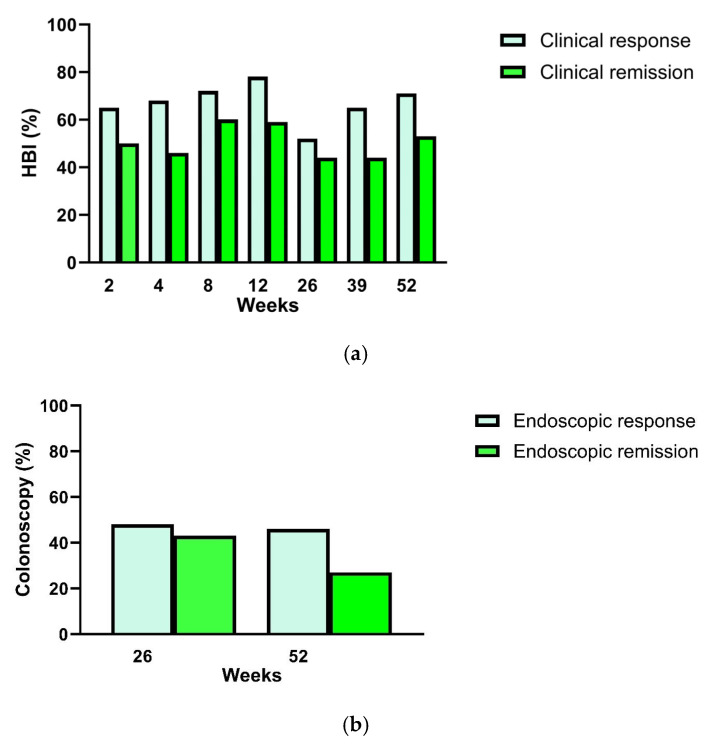

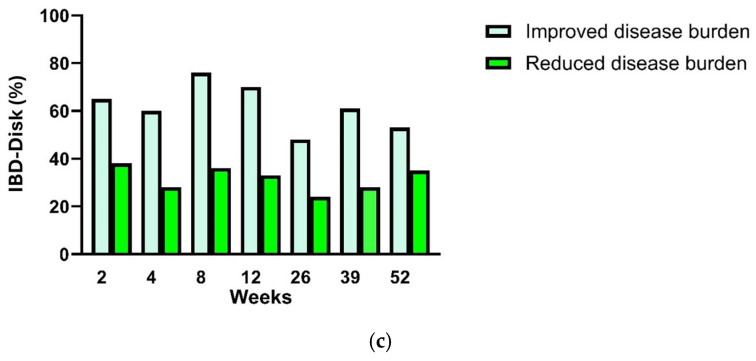
Efficacy of upadacitinib for treatment of Crohn’s disease patients during the study period (52 wk; per protocol approach). Clinical response and clinical remission were assessed by HBI (**a**), endoscopic disease activity by SES-CD score (**b**), and patient-reported outcomes (PROs) by IBD-Disk (**c**). HBI, Harvey–Bradshaw Index; IBD-Disk, inflammatory bowel disease disk, SES-CD score, Simple Endoscopic Score for Crohn’s Disease.

An endoscopic response was defined as a decrease in the SES-CD of more than 50% from baseline or for patients with an SES-CD of 4 or less at baseline, a decrease of ≥2 points from baseline (Figure 2).

Clinical remission in terms of HBI was defined as a HBI score ≤ 4.

Endoscopic remission was defined as an SES-CD of 4 or less, a decrease of at least 2 points from baseline.

Clinical response in terms of HBI was defined as a decrease in HBI score ≥ 3 points.

The Visual Analog Scale (IBD-Disk) contains ten component subscales: abdominal pain, bowel control, interpersonal interactions, education/work, sleep, energy, emotions, body image, sexuality, and joint pain. Totals score range from 0 to 100, with higher scores indicating an impaired health-related quality of life.

Reduced Disease Burden in terms of CED-Disk was defined as a decrease of 70% or a CED-Disk Score of ≤15 (change from baseline in Visual Analog Scale).

Improved Disease Burden in terms of CED-Disk was defined as a decrease of 30% or a CED-Disk Score of ≤15 (change from baseline in Visual Analog Scale).

An endoscopic response was defined as a decrease in the SES-CD of more than 50% from baseline (or for patients with an SES-CD of 4 or less at baseline, a decrease of ≥2 points from baseline).

Clinical remission in terms of HBI was defined as a HBI score ≤ 4.

Endoscopic remission was defined as an SES-CD of 4 or less, a decrease of at least 2 points from baseline.

Clinical response in terms of HBI was defined as a decrease in HBI score ≥ 3 points.

Reduced Disease Burden in terms of CED-Disk was defined as a decrease of 70% or a CED-Disk Score of ≤15 (change from baseline in Visual Analog Scale).

Improved Disease Burden in terms of CED-Disk was defined as a decrease of 30% or a CED-Disk Score of ≤15 (change from baseline in Visual Analog Scale).

## 4. Discussion

The results of this prospective multicenter real-world study demonstrate that upadacitinib represents a promising therapeutic option for biologic-experienced, anti-TNF-pretreated patients with CD with regard to the assessment of clinical disease activity, endoscopic disease activity, and PROs during induction and maintenance therapy through week 52. The data confirm and extend the findings of the current literature in highly refractory populations, that upadacitinib can effectively induce clinical and endoscopic remission and can improve PROs analyzed by IBD-Disk. The treatment of moderate-to-severe CD in patients with multiple prior therapies remains one of the greatest therapeutic challenges in gastroenterological practice, as the risk of complications and treatment failure increases with each therapeutic attempt and with disease duration [2,14,15]. The patient cohort examined in this study reflects a highly refractory population frequently encountered in clinical practice: all patients had received at least one anti-TNF therapy prior to enrolment and were generally treated with multiple biologics and conventional immunomodulators.

Compared to large, controlled registration trials (U-EXCEL, U-EXCEED, U-ENDURE) [2,16] in which, during the maintenance phase, only approximately 25% of patients were pretreated with biologics and 75% were biologic-naïve, the present cohort examined a substantially higher proportion of patients with multiple prior therapies (75% with ≥3 prior biologics), reflecting a real-world cohort which significantly differs from the patient cohort of registration trials. This selection is clinically significant, as current network meta-analyses demonstrate a gradual decline in efficacy with increasing numbers of prior biologic failures: patients with fewer prior therapies achieved significantly higher rates of clinical and endoscopic remission (weeks 12 and 52) than those with multiple biologic exposure [16]. This trend was similarly demonstrated in Phase 3 trials for adalimumab (CHARM trial), ustekinumab (UNITI-1/2), vedolizumab (GEMINI 2/3), and risankizumab (ADVANCE, MOTIVATE, FORTIFY) [17,18,19,20,21].

In our cohort of anti-TNF-experienced patients with multiple prior therapies, endoscopic response and endoscopic remission were demonstrated in 48% and 43% of patients, respectively, at week 26, and in 46% and 27%, respectively, at week 52. The primary endpoint of endoscopic treatment response was defined in the registration trials (U-EXCEL, U-EXCEED) as a decrease in SES-CD of more than 50% from baseline or, for patients with a baseline SES-CD of 4 or less, a decrease of ≥2 points from baseline. In these trials, endoscopic response was demonstrated at week 12 in 45.5% (U-EXCEL) and 35% (U-EXCEED) of patients, and at week 52, in 40% of patients. Endoscopic remission (secondary endpoint, U-EXCEL, U-EXCEED), defined as a SES-CD of 4 or less, a decrease of at least 2 points from baseline, and no subscore greater than 1 in any individual segment, was achieved at week 12 in 29% (U-EXCEL) and 19% (U-EXCEED) of patients, and at week 52 in 29% of patients [2]. The endoscopic remission rates of 43% observed at week 26 in this study actually exceed the results of the registration trials, which may be attributable to differences in endoscopic remission definitions and patient selection, and demonstrate the high efficacy of upadacitinib in anti-TNF-pretreated CD patients.

Clinical response and remission rates assessed by HBI at week 12 in this study were 78% and 59%, respectively; at week 26, 52% and 44%; and at week 52, 71% and 53%. Although the HBI correlates less well with endoscopic activity than composite scores, it remains an important clinical instrument for assessing symptomatic response [22].

When incorporating PROs using the quality-of-life score (IBD-Disk), an IDB and an RDB were observed in 70% and 33% of patients with Crohn’s disease at week 12, 48% and 24% at week 26, and 53% and 35% at week 52, respectively. These data are particularly relevant, as patients with suboptimal disease control demonstrate significantly lower quality-of-life scores, and the integration of PROs is increasingly recognized as an important component of comprehensive therapeutic assessment [23,24]. To date, there are only a few reports on the application of the IBD-Disk in IBD patients for the assessment of PROs. Singh et al. differentiated between high and low disease burden according to the IBD-Disk Score (<30 vs. ≥30). For comparability, we adopted this analysis and observed concordant results (see Supplemental Table S1) [13].

In the registration trials U-EXCEL and U-EXCEED (week 12), 49.5% and 38.9% of patients achieved clinical remission under 45 mg upadacitinib once daily, as defined by CDAI criteria (<150 points, primary endpoint). In the Phase III maintenance study U-ENDURE, 47.6% of patients achieved clinical CDAI remission under upadacitinib 30 mg at week 52. In both induction trials, clinical response, defined as a CDAI reduction of at least 100 points (CR-100) from baseline, was achieved in 57% (U-EXCEL) and 51% (U-EXCEED) of patients, and 51.2% at week 52. Stool frequency/abdominal pain (SF/APS) remission, examined as a secondary endpoint, was defined as an average daily frequency of very soft or liquid stools ≤ 2.8 and an average daily abdominal pain score ≤ 1.0, with both values not worse than baseline. This was achieved in U-EXCEL in 50.7% and in U-EXCEED in 39.8% of upadacitinib-treated patients. At week 52 under upadacitinib 30 mg, 46.4% of patients achieved SF/APS remission [2]. Notably, upadacitinib demonstrated significant improvements in fatigue, quality of life, and work productivity as early as week 4, which were sustained through week 52. This rapid improvement in PROs is clinically significant because fatigue, abdominal discomfort, and fecal incontinence are among the most frequently reported symptoms substantially impairing quality of life [23].

Currently, limited prospective data exist regarding the efficacy of upadacitinib in CD patients with prior anti-TNF exposure. The present study contributes to the growing evidence based on this population. Clinical remission rates in our prospective study based on HBI were comparable to those reported in international retrospective real-world cohorts. Friedberg et al. examined clinical treatment response following therapy initiation in anti-TNF-pretreated Crohn’s disease patients during the induction phase and demonstrated clinical treatment response (HBI decrease ≥ 3 points) in 77% (13/17) of patients and clinical remission (HBI < 5) in 71% (12/17) of patients at week 8 [25]. Danso et al. examined clinical outcomes in a retrospective real-world cohort study of patients with multiple prior therapies (64% with ≥3 biologic failures) and demonstrated clinical remission (HBI < 4) in 50% (113/227) and clinical treatment response (HBI reduction ≥3 from baseline) in 67% (151/227) of patients at 12 weeks following therapy initiation. Endoscopic remission was observed in 6% (3/46, SES-CD ≤ 3) and endoscopic treatment response (≥50% reduction in SES-CD) in 5% (2/43) of patients. Six months after therapy initiation, 45% (77/172) of patients were in clinical remission and 12% (8/66) in endoscopic remission [26]. In additional retrospective cohort studies, depending on prior therapies and definitions of response/remission endpoints, clinical response rates at 12 weeks ranged from 53 to 85%, clinical remission rates from 43 to 64%, endoscopic response rates from 47 to 50%, and remission rates from 38 to 43; by week 24 maintenance, clinical remission was demonstrated in 43–56% of patients and endoscopic remission in 43% of patients [27,28,29,30,31].

In our study, adverse events were observed in 11% of patients (4% lymphopenia, 7% skin disease), with no SAEs reported. These data are consistent with recent meta-analyses evaluating upadacitinib in CD, which have demonstrated a modestly increased, dose-dependent infection risk, particularly for herpes zoster. A comprehensive 2025 meta-analysis of 2611 IBD patients demonstrated that upadacitinib did not increase the overall risk for serious adverse events [32]. However, elevated rates of neutropenia, creatine kinase elevation, and acne were observed, whereas anemia and arthralgias were reduced. A further systematic review in 2025 with 9547 patients confirmed dose-dependent risks for hepatic abnormalities, neutropenia, and acne, but found no significant associations with non-melanoma skin cancer, cardiovascular events (MACE), or venous thromboembolism (VTE) [33,34].

This study has several limitations, including its prospective but non-randomized design, lack of blinding, and relatively small cohort size. Not all patients completed assessments at every timepoint due to treatment discontinuations and missed clinic visits, resulting in incomplete data for some parameters and timepoints. Longer follow-up data are required to assess long-term safety and durability of response, particularly regarding rare but serious adverse events. The heterogeneity of endoscopic definitions across studies complicates direct comparisons between cohorts. After the induction phase, the upadacitinib label allows IBD patients to be treated with 15 mg or 30 mg daily during the maintenance phase. In our cohort, all patients received 30 mg of upadacitinib daily, as specified in the study protocol. This higher dose was chosen due to the inclusion of biologic-experienced patients; however, it remains unclear whether our promising results can be extrapolated to the lower daily maintenance dose of upadacitinib.

## 5. Conclusions

In summary, this prospective multicenter real-world study confirms the efficacy and safety of upadacitinib in anti-TNF-pretreated CD patients with multiple prior biologic therapies. The achieved clinical and endoscopic remission rates as wells as PROs are remarkable despite the highly refractory nature of our cohort. The systematic assessment of patient-reported outcomes using the IBD-Disk allows for comprehensive evaluation of important clinical domains, such as arthralgia and social impairment, and enables the demonstration of therapeutic response in these dimensions. The favorable safety profile without SAEs in this cohort supports the use of upadacitinib as an important therapeutic option in the sequential treatment of refractory CD. These results underscore the importance of individualized treatment strategies and continuous monitoring to optimize outcomes in this challenging patient population.

## Figures and Tables

**Table 1:** Baseline characteristics of patients with Crohn’s disease (CD) treated with upadacitinib. Anti-TNF = Infliximab, Adalimumab; CD, Crohn’s disease; BMI, Body Mass Index; n, number.

Baseline Characteristics	Value
**Age at induction**, median (min–max), years	37 (25–64)
**Age at initial diagnosis**, median (min–max), years	20 (12–59)
**Median duration of disease**, (IQR), years	15 (9–18)
**Male sex**, ***n*** (%)	13 (46)
**BMI**, median (IQR), kg/m^2^	24 (22–27)
**Positive smoking status**, ***n*** (%)	6 (21)
**Location of disease**, ***n*** (%):	
L1, ileum	6 (21)
L2, colon	5 (18)
L3, ileocolon	17 (61)
**if applicable L4, upper GI tract**	3 (11)
**Disease behavior**, ***n*** (%):	
B1, inflammatory (non-stricturing/non-penetrating)	1 (4)
B2, stricturing	18 (64)
B3, penetrating	9 (32)
if applicable: Perianal disease	13 (46)
**Ileostomy**, ***n*** (%)	2
**Previous failure to biologic agents**, ***n*** (%)	
Anti-TNF	28 (100)
Vedolizumab	15 (54)
Ustekinumab	20 (71)
**No. of previous failures of biological therapy**, ***n*** (%)	
0	0 (0)
1	3 (11)
2	4 (14)
3	21 (75)
**No. of previous failures of immunosuppressants**, ***n*** (%):	
Azathioprine/6-mercaptopurine	15 (54)
MTX	6 (21)

**Table 2:** Primary and key secondary end points for upadacitinib as induction and maintenance therapy, according to EMA/FDA requirements (per protocol approach). WK, week; CI, confident interval; HBI, Harvey–Bradshaw Index; IBD-Disk, inflammatory bowel disease disk; SES-CD score, simple endoscopic score for Crohn’s Disease.

Endpoints			Induction		Maintenance				
			2 wk	4 wk	8 wk	12 wk	26 wk	39 wk	52 wk
Primary end points, % (95% CI)									
Endoscopic response						48 (44–52)		46 (21–72)	
Clinical remission (HBI)	50 (32–68)	46 (29–65)	60 (41–77)		59 (41–75)	44 (27–63)	44 (25–66)	53 (47–58)	
Secondary end points, % (95% CI)									
Endoscopic remission						43 (40–48)		27 (10–57)	
Clinical response (HBI)	65 (46–81)	68 (48–83)	72 (52–86)		78 (59–89)	52 (33–70)	65 (41–83)	71 (62–72)	
Reduced Disease Burden (IBD-Disk)	38 (37–43)	28 (28–34)	36 (34–41)		33 (32–39)	24 (24–31)	28 (27–36)	35 (33–43)	
Improved Disease Burden (IBD-Disk)	65 (60–67)	60 (55–62)	76 (69–76)		70 (65–71)	48 (45–52)	61 (54–64)	53 (47–58)	

**Table 3:** Upadacitinib in induction and maintenance therapy. Wk, weeks; SES-CD score, Simple Endoscopic Score for Crohn’s Disease; CRP, C-reactive protein; HBI, Harvey–Bradshaw Index; IBD-Disk, inflammatory bowel disease disk. Where applicable, the number of patients with evaluable data is indicated as *n*/N, where *n* represents the number of patients with completed assessment and N represents the total number of enrolled patients. Reasons for missing data include patient missed clinic visits or incomplete examination documentation.

	Baseline		Induction								Maintenance					
	0 wk		2 wk		4 wk		8 wk		12 wk		26 wk		39 wk		52 wk	
		n/N	N	n/N	N	n/N	N	n/N	N	n/N	N	n/N	N	n/N	N	n/N
Patients	28		28		28		28		28		24		19		16	
Withdrawal from treatment	0		0		0		0		1		4		9		12	
SES-CD Score	9 (6–17)	27/28									6 (2–12), *p* = 0.14	19/24			5 (1–12), *p* = 0.005	8/16
Fecal calprotectin, Median (IQR), µg/g	350 (100–1280)	26/28							184 (58–809), *p* = 0.157	23/28	289 (88–723), *p* = 0.181	21/24	114 (29–870), *p* = 0.05	9/19	214 (17–449), *p* = 0.875	12/16
Laboratory parameters, Median (IQR)																
Hemoglobin, g/dL	12.7 (11.9–13.9)	28/28							11.9 (11–12.8), *p* = 0.004	28/28	12.2 (10.9–13.5), *p* = 0.282	24/24	11.7 (10.9–13.4), *p* = 0.423	16/19	11.2 (10.7–13), *p* = 0.066	14/16
Leukocyte count, ×10^9^/L	9.1 (6.4–11.2)	28/28							6.9 (5.2–9.8), *p* = 0.017	28/28	7.1 (5.2–9.4), *p* = 0.178	24/24	6.7 (5.5–10.4), *p* = 0.222	16/19	6.4 (5.4–8.2), *p* = 0.286	14/16
CRP, mg/L	0.6 (0.5–1.4)	28/28							0.5 (0.5–0.7), *p* = 0.379	28/28	0.5 (0.5–1.2), *p* = 0.205	24/24	0.5 (0.5–0.7), *p* = 0.035	16/19	0.5 (0.5–0.6), *p* = 0.010	14/16
HBI, Median (IQR)	9 (5–14)	28/28	5 (0–7), *p* < 0.001	26/28	5 (0–7), *p* < 0.001	25/28	3 (1–10), *p* = 0.006	25/28	3 (1–8), *p* = 0.002	27/28	5 (2–10), *p* = 0.033	23/24	5 (2–8), *p* = 0.010	16/19	4 (1–8), *p* = 0.035	14/16
IBD-Disk, Median (IQR)	53 (23–73)	28/28	23 (4–47), *p* < 0.001	26/28	25 (3–58), *p* < 0.001	25/28	19 (5–41), *p* = 0.006	25/28	2 (0–7), *p* = 0.002	27/28	40 (10–60), *p* = 0.033	23/24	35 (14–51), *p* = 0.023	16/19	19 (10–38); *p* = 0.026	14/16
Abdominal pain	5 (1–8)	28/28	3 (0–6), *p* = 0.006	26/28	4 (0–6), *p* = 0.002	25/28	1 (0–5), *p* = 0.002	25/28	2 (0–7), *p* = 0.016	27/28	2 (0–6), *p* = 0.013	23/24	2 (0–5), *p* = 0.011	16/19	2 (0–4), *p* = 0.016	14/16
Stool control	5 (0–9)	28/28	2 (0–5), *p* < 0.001	26/28	0 (0–0), *p* < 0.001	25/28	1 (0–4), *p* = 0.001	25/28	1 (0–4), *p* = 0.005	27/28	1 (0–6), *p* = 0.038	23/24	0 (0–2), *p* = 0.009	16/19	2 (0–4), *p* = 0.314	14/16
Interpersonal interactions	3 (0–7)	28/28	2 (0–5), *p* = 0.047	26/28	0 (0–0), *p* = 0.176	25/28	0 (0–4), *p* = 0.006	25/28	0 (0–5), *p* = 0.07	27/28	4 (0–8), *p* = 0.944	23/24	4 (0–8), *p* = 0.815	16/19	1 (0–3), *p* = 0.186	14/16
Education/Work	6 (0–10)	28/28	0 (0–0), *p* = 0.003	26/28	0 (0–0), *p* = 0.114	25/28	0 (0–5), *p* = 0.007	25/28	1 (0–5), *p* = 0.048	27/28	4 (0–8), *p* = 0.337	23/24	2 (0–6), *p* = 0.012	16/19	0 (0–5), *p* = 0.132	14/16
Sleep	6 (3–8)	28/28	3 (0–5), *p* < 0.001	26/28	3 (0–6), *p* = 0.001	25/28	3 (0–6), *p* < 0.001	25/28	4 (0–7), *p* = 0.018	27/28	4 (0–6), *p* = 0.015	23/24	4 (0–6), *p* = 0.109	16/19	2 (0–4), *p* = 0.017	14/16
Energy	7 (4–10)	28/28	4 (0–7), *p* < 0.001	26/28	5 (0–7), *p* = 0.01	25/28	5 (0–6), *p* = 0.004	25/28	4 (0–7), *p* = 0.002	27/28	5 (2–8), *p* = 0.147	23/24	5 (2–8), *p* = 0.299	16/19	3 (0–6), *p* = 0.025	14/16
Emotions	5 (3–7)	28/28	0 (0–5), *p* < 0.001	26/28	0 (0–6), *p* = 0.003	25/28	0 (0–5), *p* < 0.001	25/28	2 (0–5), *p* = 0.006	27/28	5 (0–8), *p* = 0.123	23/24	3 (1–7), *p* = 0.076	16/19	2 (0–5), *p* = 0.021	14/16
Body image	4 (0–7)	28/28	0 (0–5), *p* = 0.01	26/28	0 (0–0), *p* = 0.084	25/28	1 (0–4), *p* = 0.017	25/28	0 (0–5), *p* = 0.097	27/28	3 (0–6), *p* = 0.364	23/24	4 (0–7), *p* = 0.916	16/19	2 (0–5), *p* = 0.166	14/16
Sexuality	5 (0–8)	28/28	0 (0–5), *p* < 0.001	26/28	0 (0–0), *p* = 0.011	25/28	0 (0–5), *p* = 0.001	25/28	1 (0–5), *p* = 0.016	27/28	3 (0–6), *p* = 0.081	23/24	5 (0–7), *p* = 0.048	16/19	0 (0–3), *p* = 0.007	14/16
Joint pain	6 (0–8)	28/28	0 (0–5), *p* = 0.005	26/28	0 (0–7), *p* = 0.029	25/28	1 (0–5), *p* = 0.003	25/28	0 (0–4), *p* = 0.024	27/28	2 (0–8), *p* = 0.419	23/24	2 (0–7), *p* = 0.555	16/19	4 (0–6), *p* = 1.000	14/16
Concomitant Crohn’s disease medications																
Glucocorticoids	15 (54)	28/28							4 (15)	28/28	5 (21)	24/24	2 (11)	19/19	1 (6.3)	16/16

Pairwise comparisons of changes from baseline to each respective time point were performed using the Wilcoxon signed-rank test for paired samples.

**Table 4:** Overview of adverse events after initiating upadacitinib in Crohn’s disease patients during a 1-year study period.

Adverse Events	*n* (%)
Any adverse event	
Severe adverse event	0 (0)
Serious adverse event	0 (0)
Death from any cause	0 (0)
Adverse events of special interest	3 (11)
Serious infection	0 (0)
Opportunistic infection	0 (0)
Herpes zoster infection	0 (0)
Tuberculosis	0 (0)
Lymphopenia	1 (4)
Skin disease	2 (7)
Adjudicated cardiovascular events	0 (0)
Adjudicated thrombotic events	0 (0)
Cancer of any type	0 (0)

**Table 5:** Reasons for discontinuing therapy with upadacitinib.

Reasons	*n* (%)
Primary non-response	7 (25)
Adverse events	3 (11)
Skin disease	2 (7)
Lymphopenia	1 (4)
Others	2 (7)

## Data Availability

The datasets presented in this article are not readily available due to privacy and ethical restrictions.

## Supplementary Materials

The following supporting information can be downloaded at: https://www.mdpi.com/article/10.3390/diseases14020054/s1, Table S1: Disease burden IBD-Disk 30.
