# Supplementary material for: Prospective Bi-Centric Real-World Outcomes of Upadacitinib in Biologic-Experienced Patients with Crohn’s Disease
Source: Diseases. 2026 Feb 1;14(2):54. doi: 10.3390/diseases14020054 (PMC12939649; doi:10.3390/diseases14020054)
Supplement: Supplementary file 1 [file diseases-14-00054-s001.zip › diseases-4054393-supplementary.pdf]

Supplemental Table S1: Disease burden IBD-Disk 30: Wk, weeks; IBD-Disk, inflammatory bowel disease Disk; Where applicable, the number of patients with evaluable data is indicated as n/N, where n represents the number of patients with completed assessment and N represents the total number of enrolled patients. Reasons for missing data include patient missed clinic visits or incomplete examination documentation.

	**Baseline**		**Induction**								**Maintenance**					
	0 wk		2 wk		4 wk		8 wk		12 wk		26 wk		39 wk		52 wk	
		n/N	N	n/N	N	n/N	N	n/N	N	n/N	N	n/N	N	n/N	N	n/N
**IBD Disk ≤ 30**	9 (32)	28/28	15 (58)	26/28	14 (56)	25/28	6 (24)	25/28	15 (56)	27/28	13 (52)	23/24	9 (50)	16/19	13 (77)	14/19
